# Immunoproteasome LMP2 60HH Variant Alters MBP Epitope Generation and Reduces the Risk to Develop Multiple Sclerosis in Italian Female Population

**DOI:** 10.1371/journal.pone.0009287

**Published:** 2010-02-18

**Authors:** Michele Mishto, Elena Bellavista, Claudia Ligorio, Kathrin Textoris-Taube, Aurelia Santoro, Mara Giordano, Sandra D'Alfonso, Florinda Listì, Benedetta Nacmias, Elena Cellini, Maurizio Leone, Luigi M.E. Grimaldi, Chiara Fenoglio, Federica Esposito, Filippo Martinelli-Boneschi, Daniela Galimberti, Elio Scarpini, Ulrike Seifert, Maria Pia Amato, Calogero Caruso, Maria P. Foschini, Peter M. Kloetzel, Claudio Franceschi

**Affiliations:** 1 Department of Experimental Pathology, University of Bologna, Bologna, Italy; 2 Interdepartmental Center for Studies on Biophysics, Bioinformatics and Biocomplexity ‘L. Galvani’ (CIG), Bologna, Italy; 3 Institute of Biochemistry, Medical Faculty Charité, Berlin, Germany; 4 Department of Hematology and Oncology, Section of Anatomic Pathology, University of Bologna and Bellaria Hospital, Bologna, Italy; 5 Department of Medical Sciences and IRCAD (Interdisciplinary Research Center of Autoimmune Diseases), University of Eastern Piedmont, Novara, Italy; 6 Department of Biopathology & Biomedical Methodologies, University of Palermo, Palermo, Italy; 7 Department of Neurological and Psychiatric Sciences, University of Florence, Florence, Italy; 8 Clinica Neurologica, Ospedale Maggiore della Carità, Novara, Italy; 9 U.O. Neurologia Fondazione, Istituto San Raffaele “G.Giglio” di Cefalù, Cefalù (PA), Italy; 10 Department of Neurological Sciences, University of Milan, IRCCS Fondazione Ospedale Maggiore Policlinico, Milan, Italy; 11 Institute of Experimental Neurology (INSPE), and Department of Neurology, San Raffaele Scientific Institute, Milan, Italy; Julius-Maximilians-Universität Würzburg, Germany

## Abstract

**Background:**

Albeit several studies pointed out the pivotal role that CD4+T cells have in Multiple Sclerosis, the CD8+ T cells involvement in the pathology is still in its early phases of investigation. Proteasome degradation is the key step in the production of MHC class I-restricted epitopes and therefore its activity could be an important element in the activation and regulation of autoreactive CD8+ T cells in Multiple Sclerosis.

**Methodology/Principal Findings:**

Immunoproteasomes and PA28-αβ regulator are present in MS affected brain area and accumulated in plaques. They are expressed in cell types supposed to be involved in MS development such as neurons, endothelial cells, oligodendrocytes, macrophages/macroglia and lymphocytes. Furthermore, in a genetic study on 1262 Italian MS cases and 845 controls we observed that HLA-A*02+ female subjects carrying the immunoproteasome LMP2 codon 60HH variant have a reduced risk to develop MS. Accordingly, immunoproteasomes carrying the LMP2 60H allele produce *in vitro* a lower amount of the HLA-A*0201 restricted immunodominant epitope MBP_111–119_.

**Conclusion/Significance:**

The immunoproteasome LMP2 60HH variant reduces the risk to develop MS amongst Italian HLA-A*02+ females. We propose that such an effect is mediated by the altered proteasome-dependent production of a specific MBP epitope presented on the MHC class I. Our observations thereby support the hypothesis of an involvement of immunoproteasome in the MS pathogenesis.

## Introduction

Multiple Sclerosis (MS) is the most common autoimmune disorder of the central nervous system (CNS). It is characterized by multifocal areas of demyelization (plaques), chronic inflammation and damage to oligodendrocytes and neurons. The cause of MS is still unknown and disease pathways are poorly understood. However, the association of HLA-DRB1*15 and other HLA class I (*e.g.* HLA-A*02 and HLA-A*03) and class II alleles, the presence of autoreactive T lymphocytes together with other inflammatory cells and cytokines in active MS lesions suggest an autoimmune pathogenesis. Accordingly, the experimental autoimmune encephalomyelitis (EAE), a classical mouse model for MS, can be induced by the administration of myelin antigens or CD4+ and CD8+ T lymphocytes specific for those antigens [1], [2], [3]. On the basis of recent results on EAE, it has been proposed that the first bout of the disease is mediated by CD8+ T cells while the first relapse and further disease are mediated by CD4+ T cells through different mechanisms such as antigen release and epitope spreading [4].

The antigen bound on Major Histocompatibility Complex class I (MHC-I) and presented to the T cell receptor (TCR) of CD8+ T cells, is normally produced by proteasomes. One of the proteasome isoforms, known as immunoproteasome, enhances the generation of specific antigenic epitopes [5] and its role in neurodegenerative diseases has recently been described [6], [7]. Immunoproteasome differs from the constitutive proteasome by the β subunits with catalytic activity [β1, β2, β5 into constitutive proteasome and Low Molecular Mass Polypeptide 2 (LMP2), Multicatalytic Endopeptidase Complex Subunit (MECL-1) and LMP7 into immunoproteasome] [5]. Immunoproteasome assembly can be induced by IFN-γ, which also stimulates the expression of proteasome activator-αβ (PA28-αβ). This regulatory complex binds to the end of the 20S proteasome core, increasing the cleavage rate in an ATP-independent manner and affecting the quality of protein digestion [5], [8]. Moreover, a role for immunoproteasomes has been suggested in some autoimmune diseases such as ankylosing spondylitis and psoriasis because of the association of a polymorphism of its LMP2 subunit to the those pathologies [9], [10], [11]. This particular polymorphism is a non-conservative nucleotide base pair change at amino acid position 60 (in exon 3), resulting in two alleles, arginine (R) or histidine (H). It modifies the susceptibility of peripheral blood mononuclear cells to TNF-α induced-apoptosis in elderly donors [12], as well as the proteasome activity in brain protein extracts from Alzheimer patients and non-demented elderly [7].

In this study, we investigated the presence of immunoproteasome in the CNS of MS patients and whether its LMP2 polymorphism might be involved in MS onset. The data we obtained indicates that immunoproteasomes and PA28-αβ regulator are present in MS CNS and that the LMP2 60HH genotype impinges upon MS, likely in part by reducing the production of a specific HLA-A*02 restricted Myelin Basic Protein epitope (MBP_111–119_) implicated in MS pathology [13], [14], [15] as shown in *in vitro* assays and in the presence of PA28-αβ. Furthermore, these results add further evidences on the complex role of HLA-A*02 allele in the pathogenesis of this disease.

## Materials and Methods

The Ethics Committees of the University of Bologna, Florence, Empoli, Milan and Eastern Piedmont approved this study. An informed written consent to participate in this study was obtained from all participants.

### Patients and Controls

Two independent MS populations were genotyped and compared to a common Italian control population. The samples consisted of: i. 694 MS patients (mean age: 38.8+/−11.7 years) for the first MS population consecutively referred to the Department of Neurology of the University of Florence, the Hospital of Empoli, the University of Eastern Piedmont (Novara); ii. 568 MS patients (mean age: 43.4+/−12.4 years) for the second MS population referred to the Institute of Experimental Neurology and Department of Neurology - San Raffaele Scientific Institute (Milan) and the IRCCS Fondazione Ospedale Maggiore Policlinico (Milan). The two independent samples were compared to a population of 845 age- and area-matched controls (mean age: 37.8+/−12.9 years) and then grouped providing a total MS sample of 1262 patients (mean age: 41.6+/−12.3). Inclusion criteria were a definite diagnosis of MS [16], age ≥18 years and written informed consent. All controls were carefully assessed to exclude diagnosis of inflammatory disorders. DNA was extracted from peripheral blood of controls and MS patients as previously described [12].

### Materials and Chemicals

Fetal calf serum and RPMI 1640 medium were from Biowhittaker (Belgium). The restriction enzymes were from MBI Fermentas (Latvia). The peptide MBP_102–109_ (seq. 102–129 of the human classical Myelin Basic Protein (MBP) isoform 5) has the following sequence: PSQGKGRGL**SLSRFSWGA**EGQRPGFGYG-CONH2 term. In bold the sequence of the analyzed epitope MBP_111–119,_ HLA-A*0201 restricted, detected in MS patient blood and believed to be involved in MS [13], [14], [15]. The epitope (ELA)-MART1_26–35_ used in comparison with MBP_111–119_ has the following sequence: ELAGIGILTV-COOH term [17]. All peptides have been synthesized by Dr. P. Henklein (Institute of Biochemistry, Medical Faculty Charité, Berlin, Germany).

### Immunohistochemistry (IHC)

**Figure 1:**
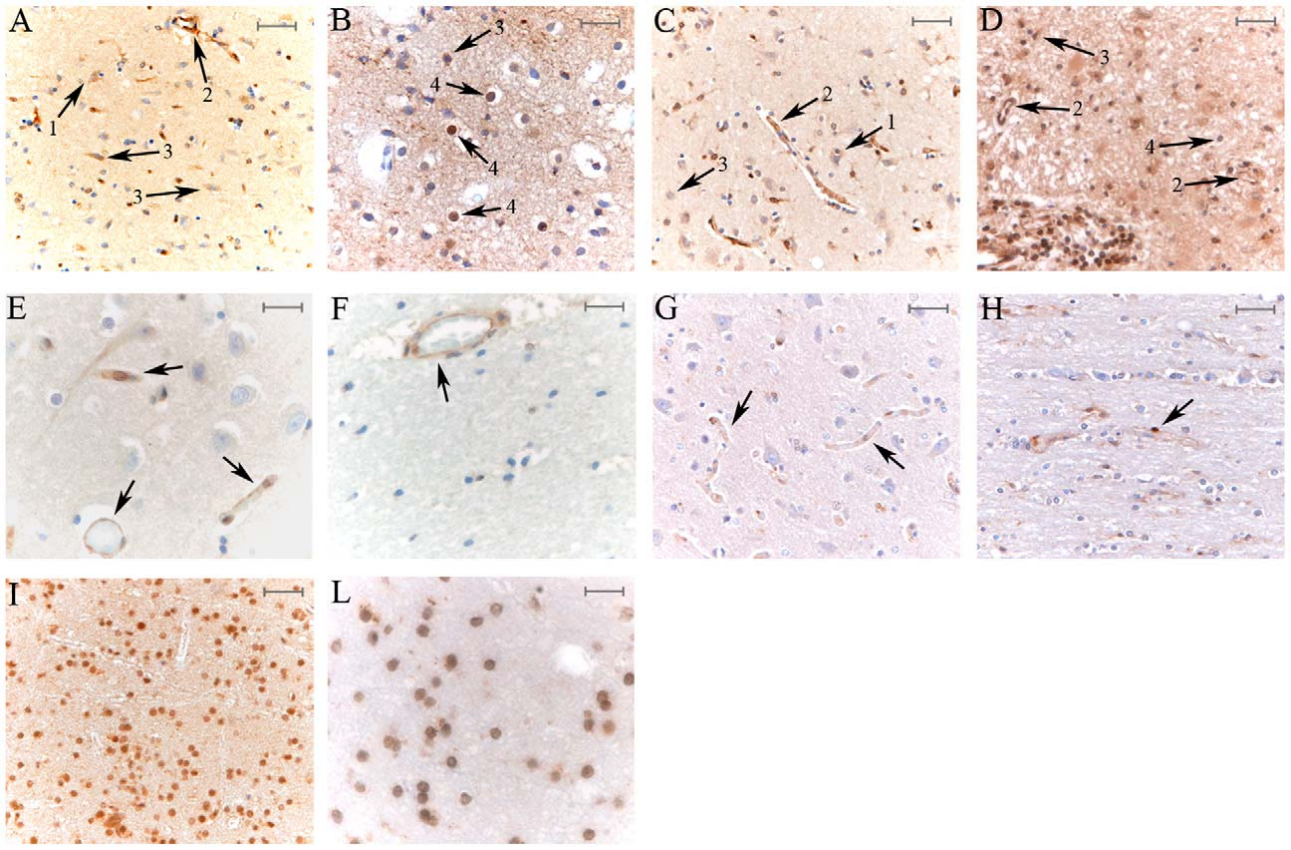
LMP2 subunit and PA28-αβ are expressed in CNS cortex and white matter of MS but not of young controls. Example of cortex (A, C) and plaque into white matter (B, D) of MS parietal lobe, stained with anti-LMP2 (A, B) and anti-PA28-α (C, D) Abs are shown. Different cell types, positive to the staining, are marked as following: 1. neurons; 2. luminal endothelial cells; 3. astrocytes; 4. oligodendrocytes (putative). Example of a parietal lobe cortex (E, G) and white matter (F, H) of young control stained with anti-LMP2 (E, F) and anti-PA28-α (G, H) Abs, which bind only luminal endothelial cells (see arrow). In (I) and (L) example of a parietal lobe white matter of a MS patient stained with anti-β1 and anti-α4 Abs are shown, respectively. As expected, all cells are positive to both stainings. The representative samples here reported derived from gender- and age-matched MS and control samples. A, C, D, F, G, H, I scale bars = 50 µm; B, E, L scale bars = 25 µm.

**Figure 2:**
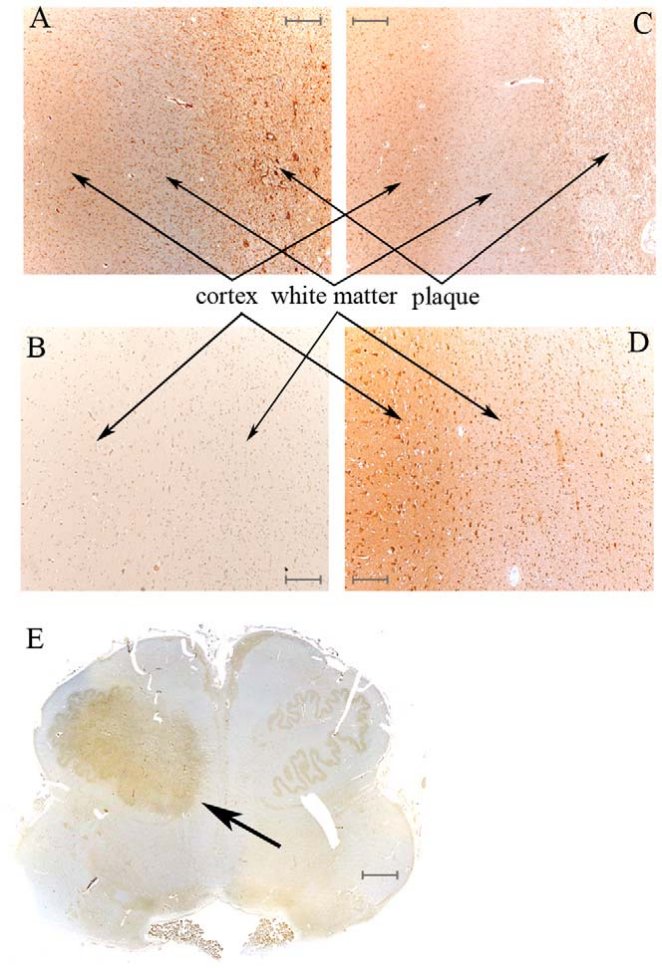
LMP2 subunit is accumulated in MS plaques. (A) Cortex, white matter and plaque of MS parietal lobe stained with anti-LMP2 Ab; an increase of cells expressing LMP2 in the plaque emerges. (B) In control parietal lobe, cortex and white matter marked with anti-LMP2 Ab show a detectable staining only in luminal endothelial cells. The labeling with anti-β1 Ab of the same MS (C) and control (D) brain area do not show any difference neither between MS and control nor between grey and white matters. (E) Rostral medulla, with MS plaque into inferior olivar nucleus (arrow), stained with anti-LMP2 Ab, which mainly binds the MS plaque. The representative samples here reported derived from gender- and age-matched MS and control samples. A-D scale bars = 300 µm; E scale bar = 3 mm.

**Figure 3:**
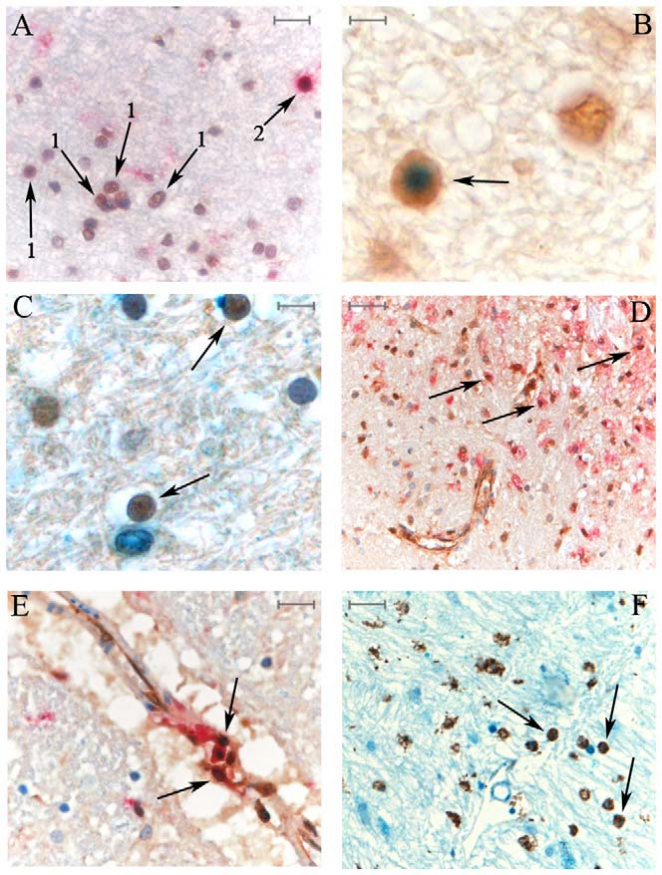
LMP2 subunit and PA28-αβ are expressed in different cell types, including oligodendrocytes, macrophages and microglial cells in MS brains. A) To discriminate among oligodendrocytes and lymphocytes, we performed a double staining with anti-LMP2 (brown) and anti- CD45RB (red) Abs in parietal lobe of MS brains. Many oligodendrocytes (only brown stained; marked with 1) and lymphocytes (brown and red stained; marked with 2) are visible. B) Oligodendrocyte identification was confirmed by staining the same area with anti-LMP2 (brown) and anti-Olig2 (blue) Abs and observing a clear labeling of oligodendrocytes for both Olig2 and LMP2 (marked by arrow). C) Oligodendrocytes (marked by arrow) express also PA28-αβ as emerged by the double staining of MS parietal lobe with anti-PA28-α (blue) and anti-Olig2 (brown). A double staining for LMP2 (brown) and CD68 (red) verified that microglia/macrophages (marked with arrows) express the immunoproteasome subunit in parenchyma (D) and perivascular (E) parietal lobe of MS brains. F) Microglia/macrophages (marked with arrows) also express PA28-αβ as revealed by the double staining with anti-PA28-α (blue) and anti-CD68 (brown) Abs. A, E scale bars = 25 µm; B, C scale bars = 10 µm. D, F scale bars = 50 µm.

In the present study we described “immunoproteasomes” as any iso-form of proteasome that contains immunosubunits, thereby also including intermediate-types [18], [19]. IHC assays were performed on parietal lobes and rostral medulla (with MS plaques) of three MS patients (2 males, 37 and 49 years old; 1 female, 49 years old) and two young controls (females, 21 and 42 years old), provided by the Department of Hematology and Oncology, “L. and A. Seragnoli” Section of Anatomic Pathology, University of Bologna at Bellaria Hospital, Bologna, Italy. The IHC showed in Figure 1, 2 and 3 referred to autopsy samples derived from MS and control donors (respectively 49 and 42 years old) died by lung embolus and gastric hemorrhage, respectively. Both autopsies were carried out after 24 h from the death and almost all brain was included for histological examination and diagnostic definition of the disease. For the present study we selected brain areas carrying the histopathological features of active chronic plaques. These plaques, included for our immunohistochemical examination, were characterized by area of myelin reduction containing numerous macrophages with clear and foamy cytoplasm, positive with acid periodic Schiff staining (PAS) after diastase digestion. The same macrophages contained myelin degradation products, which reacted with the Luxol Fast Blue (LFB) staining and were mainly located in the peripheral part of the plaque as previously described [20]. The presence of macrophages and microglial cells has been evidenced by anti-CD68 immunostaining. In addition small venules, surrounded by mature lymphocytes, were present within the plaques. The lymphocyte population, characterized at the time of the diagnosis, consisted mainly of mature T cells, CD8 positive. The other MS and controls samples were biopsies obtained during surgery to investigate the presence of possible cancers. In the brain of subjects classified as MS patients characteristic MS lesions, were identified, instead of cancer markers; in contrast, neither cancer nor MS plaques were observed in the control sample.

Immunostaining was performed as previously described [7] adopting a dilution 1∶100 of LMP2 and 1∶50 of LMP7 antibodies (Abs) (Affinity- Biomol International, Plymouth Meeting, PA, USA). In addition, mouse monoclonal Ab to human 20S proteasome subunit α4 (diluted 1∶25; Affinity- Biomol International, Plymouth Meeting, PA, USA) and rabbit polyclonal PA28α (diluted 1∶50; Affinity- Biomol International, Plymouth Meeting, PA, USA) were used. Double stainings with Abs anti-LMP2 and anti-CD68 (Dako, Carpintera, CA, USA, diluted 1∶150, clone KP1 that recognizes macrophages/microglial cells), anti LMP2 and anti-CD45RB (Dako, Carpintera, CA, USA, diluted 1∶600; Abs from clones 2B11 + PD7\26 that recognizes antigens clustered as CD45RB specific for mature lymphocytes) were performed to identify microglia/macrophages or to distinguish between oligodendrocytes and lymphocytes. In addition, oligodendrocytes identification was also carried out by double immunostaining with anti-LMP2 and anti-Olig2 (Chemicon, Billerica, MA, USA; dilution 1∶300; polyclonal rabbit that recognizes the transcription factor Olig2 [21] specific for oligodendrocytes) as well as anti-PA28-α and anti-Olig2. Double immunostainings with anti-LMP2 and anti-CD45RB or anti-CD68 were performed according to the following procedure: after biotinylated goat anti-polyvalent, two types of streptavidin were used, the first conjugated with peroxidase (Lab VISION Ultra vision Large Detection System Anti-polyvalent HRP, Fremont, CA, USA) and the second with alkaline phosphatase (DakoCytomation ChemMate Detectin Kit, Alkaline Phosphatase/RED, Rabbit/Mouse K5005, Carpintera, CA, USA) and two different chromogens: 3,3′-diaminobenzidine (Dako Liquid DAB-substrate- Chromogen system K3468, Carpintera, CA, USA) for streptavidin peroxidase and the fast red Working solution (CHROM) (Dako, Carpintera, CA, USA) for alkaline phosphatase. Double immunostainings with anti-LMP2/anti-Olig2 and with anti-PA28-α/anti-Olig2 were performed following the previous procedure but employing as second chromogen Perma\Blue AP Chromogen (Diagnostic Biosystems, Pleasonton, CA, USA) for alkaline phosphatase.

### Cell Cultures

Lymphoblastoid cell lines (LcLs) are human B lymphocytes immortalized with Epstein Barr virus (EBV) which mainly express immunoproteasomes, as previously reported [22]. T2 cell line is a human T cell leukemia/B cell line hybrid defective in TAP1/TAP2 and LMP2/LMP7 subunits and expressing the HLA-A*0201 allele [23]. LcLs and T2 cell lines were cultured in RPMI1640 medium supplemented with 10% FCS.

### Isolation of DNA from LcLs and Genotyping of Patients, Controls and LcLs

DNA from LcLs was collected from 15*10^6^ cells using TRIZOL reagent (Life Technologies, Paisley, UK) as previously reported [22]. DNA from donors for the genetic study was obtained from peripheral blood and genotyped for LMP2 codon 60 polymorphism, HLA-A*02 and HLA-DRB1*15 alleles. LMP2 genotyping was performed using standard PCR methods and DNA enzymatic digestions as previously described [12]. Genotyping for HLA-A*02 and HLA-DRB1*15 was performed by PCR-SSP analysis; as internal controls, regions of 330 bp of β2 microglobulin and 520 bp of MOG (Myelin Oligodendrocyte Glycoprotein) promoter were amplified.

### 20S Proteasome and PA28-αβ Purifications

PA28-αβ and 20S proteasomes were purified from 9 LcLs (genotyped for LMP2 codon 60 polymorphism) as previously described [8]. The final step of the PA28-αβ purification was the fractioning by chromatography on a phenylsepharose column of pooled material obtained from all the LCLs. Isolated PA28-αβ stimulated proteasomal activity on Suc-LLVY-MCA substrate up to five-fold and no activity was observed in controls without 20S proteasome testifying the absence of protease contamination in the PA28-αβ preparation. Final purified PA28-αβ was tested on SDS-PAGE and stained with Comassie-Blue.

### Digestion of Long Substrates, Epitope Detection and Their Quantification by Mass Spectrometry

The digestion of the synthetic peptide MBP_102–129_ by purified 20S proteasomes+/−PA28-αβ was performed in Hepes buffer in different condition. In particular, to examine the effects of LMP2 polymorphism on MBP_102–129_ degradation, 20 µg MBP_102–129_ peptide was dissolved in 100 µl buffer-1 (20 mM Hepes pH 7.8, 2 mM MgAcetate, 1 mM dithiothreitol) and incubated with 0.5 µg 20S proteasome or dissolved in 100 µl buffer-2 (20 mM Hepes pH 7.8, 1 mM dithiothreitol) and incubated with 0.25 µg 20S proteasome+3 µl PA28-αβ. To examine the effects of PA28-αβ on MBP_102–129_ degradation, 20 µg MBP_102–129_ was incubated with 0.25 µg 20S proteasome+/−3 µl PA28-αβ. Digestions were stopped by acidic inactivation and samples frozen. Samples were analyzed by mass spectrometry as previously described [22]. Briefly, they were quantified by C18 reversed phase HPLC (HP1100, Agilent) followed by an ESI-MS analyses performed online with a LCQ ion trap mass spectrometer (Thermo Fisher). Peptides were identified by their molecular masses calculated from the m/z peaks of the single or multiple charged ions and were confirmed by tandem mass spectrometry sequencing analyses. To test the absence of protease contamination in the PA28-αβ preparation, the substrate MBP_102–129_ was digested in the usual digestion solution without 20S proteasomes for 60 mins and no detectable degradation of the substrate was observed. Moreover, when 20S proteasomes were inhibited by MG132 (final concentration of 5 µM) no substrate degradation was observed, confirming the absence of other proteases in the 20S proteasome preparation. The impact of LMP2 R60H polymorphism on MBP_102–129_ degradation was tested computing the degradation rate constant (K_1_) in a reaction of first order. The Specific Production (SP) of the fragments 10–18 (MBP_111–119_ epitope) by 20S immunoproteasomes +/− PA28-αβ carrying different LMP2 60 variants was computed as following: (fragment signal)/(signal of substrate degraded) multiplied for a factor of 1000 (to facilitate the comparison by readers). The SP provided *de facto* an estimation of the epitope produced per digested substrate based on the mass spectrometry signal. Only peptide signals in samples with similar substrate degradation and less than 50% of substrate consumption were compared to minimize errors during peptide quantification and the re-entry of products in the proteasome core, consequently obtaining epitope SP by 20S proteasomes carrying different LMP2 codon 60 variants at different percentage of substrate degraded (<25%, between 25% and 35%, between 35% and 50%).

### Flow Cytometric Analysis of the Binding Affinity of Epitopes-HLA-A*0201

T2 cells (3×10^5^ cells/ml) were incubated with various concentrations of peptides MBP_111–119_ or (ELA)-MART1_26–35_ (100–0,1 µg/ml) in serum-free RPMI 1640 medium for 16h. Afterwards, cells were washed and incubated with anti-HLA-A*02 Ab-FITC and the HLA-A*02 stabilization on cell surface by bound epitope was measured by flow cytometer FACScalibur. Net fluorescence reported in Fig. S1 was computed by subtraction of fluorescence detected in control samples (with Ab and without peptides).

### Statistical Analyses

Statistical analyses of *in vitro* digestion data were performed using the tests of Monte Carlo Anova to evaluate the effect of LMP2 R60H polymorphism, *t*-student for independent samples to evaluate the effect of LMP2 60H allele and Mann-Whitney test to evaluate the effect of PA28-αβ. To compare mass spectrometry values obtained from different experiments, mass spectrometry signals were standardized within each degradation set and then compared. In each data set, homogeneity of variance was checked by Levene's test. Comparisons of genetic distributions were assessed by Monte Carlo Pearson χ^2^ test. Investigation of MS onset age was performed with Monte Carlo Anova test. Hardy Weinberg equilibrium was tested using the Pearson χ^2^ test. p<0.05 was considered statistically significant. All the analyses were implemented using SPSS software. Pair-wise linkage disequilibrium was evaluated by two linkage disequilibrium parameters, Lewontin's D2 [24] and r^2^ [25], which were both calculated in haploview (http://www.broad.mit.edu/mpg/haploview/) [26].

## Results

### Immunoproteasome Subunits Are Present in the CNS of MS Patients (and Concentrated in MS Plaques) but Not of Young Controls

Although the mechanisms of MS are still largely unknown, some cell types seem to be involved in the pathogenesis of this disease. Therefore, we first investigated if immunoproteasome and PA28-αβ subunits are expressed in MS CNS and in which cells. Grey and white matters of parietal lobe from three MS patients and two young controls were initially stained with Abs specific for constitutive proteasome (β1), immunoproteasome (LMP2 & LMP7) and PA28-α subunits. In the cortex (Fig. 1A) and in the plaques of the white matter (Fig. 1B) of MS patients, LMP2 was detected in different cell types. Similar results were obtained staining the same area with Abs anti-LMP7 (data not shown) and anti-PA28-α (Fig. 1C, D). In contrast, all stainings were negative both in cortex and white matter of the same CNS area of young controls except for luminal endothelial cells, which were positive to anti-LMP2 (Fig. 1E, F) and anti-PA28-α (Fig. 1G, H) Abs. As expected, proteasome β1 and α4 subunits were expressed in all cell types, both in MS patients (Fig. 1I & L, respectively) and in young controls (data not shown).

Moreover, LMP2 expression was predominant in MS plaques (Fig. 2A) compared to pre-plaque white matter and cortex while in the same CNS area of control only luminal endothelial cells were stained (Fig. 2B). Otherwise, β1 subunit was equally distributed in grey and white matter of MS (Fig. 2C) and control (Fig. 2D). Such an accumulation of the immunoproteasome subunit in the plaque area was even more evident in the rostral medulla of a MS patient stained with Ab anti-LMP2, which mainly spotted the plaque into inferior olivar nucleus, whereas other areas were weakly stained (Fig. 2E).

### Immunoproteasome and PA28-αβ Subunits Are Expressed in Different Cell Types in the CNS of MS Patients

In the CNS areas affected by the disease, LMP2 and PA28-αβ were expressed in different cell types such as neurons, luminal endothelial cells, astrocytes (Fig. 1A, C) and, in white matter plaques, also in (putative) oligodendrocytes (Fig. 1B, D). To verify that oligodendrocytes express LMP2 & PA28-αβ and to distinguish them from lymphocytes, we performed a double IHC staining of MS parietal lobe slices using Abs anti-LMP2 (or anti-PA28-α) and anti-CD45RB (marker of mature lymphocytes). As shown in Figure 3A, lymphocytes present in white matter were positive to anti-LMP2 and anti-CD45RB staining. In addition, small rounded nuclei, positive to anti-LMP2 and negative to anti-CD45RB Abs, corresponding to oligodendrocytes, were present. This result was further confirmed by anti-LMP2 and anti-Olig2 labeling of the same MS brain area where oligodendrocytes were positive to both LMP2 and the oligodendrocytes transcription factor Olig2, while other cells were only stained for LMP2 (Fig. 3B). Similarly, PA28-αβ was detected in oligodendrocytes, positive to both anti-PA28-α and anti-Olig2 Abs (Fig. 3C).

Furthermore, we performed IHC double staining with Abs anti-CD68 (marker of microglial cells and macrophages) and anti-LMP2 (or anti-PA28-αβ) in MS parietal lobe. Microglial cells and macrophages expressed both CD68 and LMP2 (Fig. 3D, E) as well as CD68 and PA28-αβ (Fig. 3F), proving that immunoproteasomes were present in these cell types also *in vivo* and thus extending the *in vitro* observations of Stohwasser and co-workers [27].

### The Immunodominant Epitope MBP_111–119_ Is Produced In Vitro by Immunoproteasomes and PA28-αβ Increases the Efficiency of Its Production

To test the involvement of immunoproteasome and its LMP2 R60H polymorphism in MS we investigated one of the major immunodominant self-epitopes detected in MS patient blood [13], [14], [15], *i.e.* MBP_111–119_ (HLA-A*0201-restricted). This epitope bound the MHC–I (HLA-A*0201) with a relative good affinity (Fig. S1) and is present in both the classic and golli MBP. This an important aspect because, as demonstrated in EAE, these two MBP families can play different roles in central and peripheral tolerance towards MBP-reactive CD8+ T cells [28], [29].

The 28mer peptide (MBP_102–129_), which contains the epitope MBP_111–119_, was digested *in vitro* by 20S immunoproteasomes in presence or absence of PA28-αβ, which is strongly implicated in MHC-I antigen presentation by increasing the production of some epitopes [30], [31].

**Figure 4:**
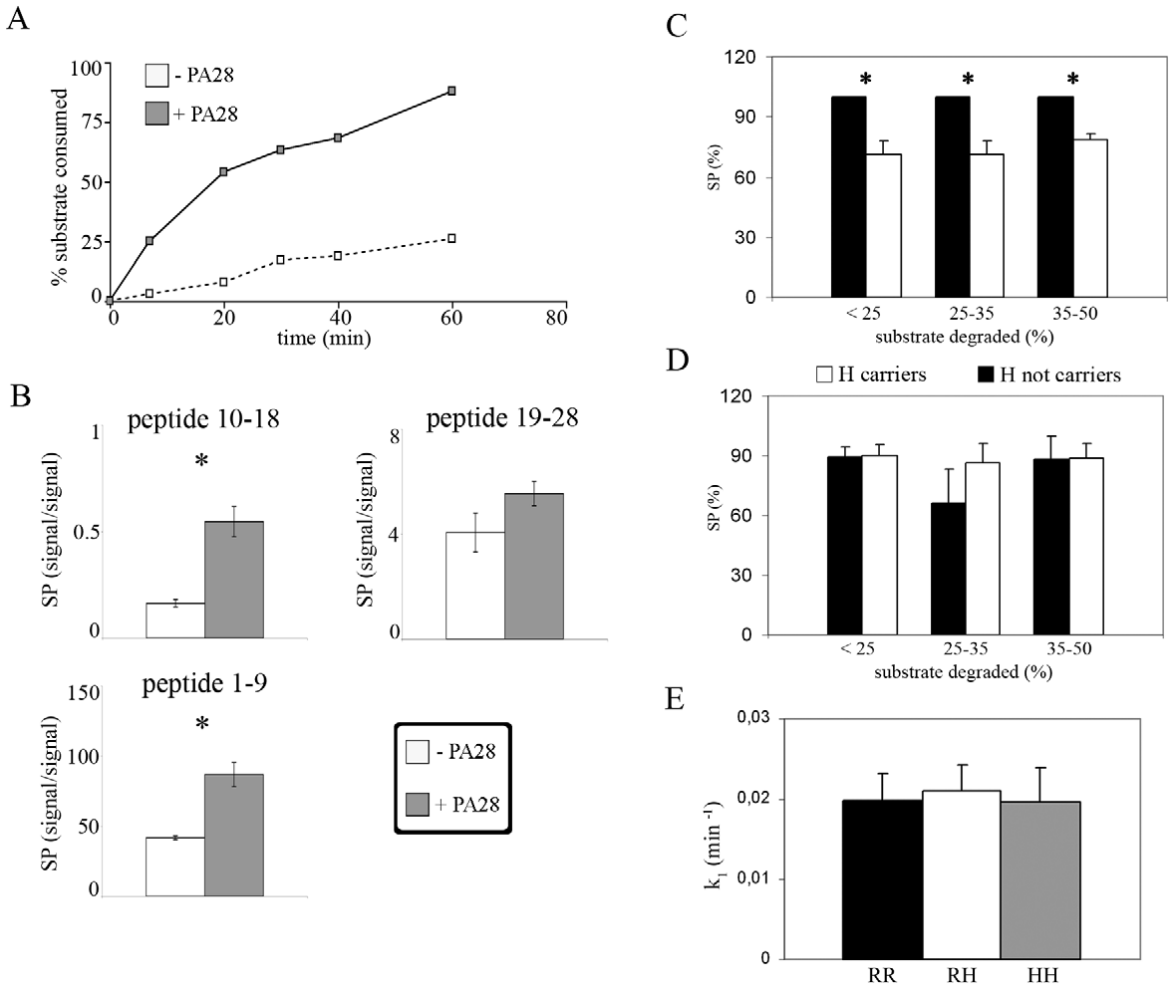
20S immunoproteasomes (+ PA28-αβ) carrying LMP2 60H allele have a lower SP of the MBP_111–119_ epitope. (A) Chart shows the time-dependent kinetics of MBP_102–129_ degradation increased in presence of PA28**-**αβ. (B) Specific production (SP) [expressed as: (peptide signal/consumed substrate signal)*1000] of the MBP_111–119_ epitope (peptide 10–18 of MBP_102–129_) and of the two flanking peptides 1–9 and 19–28 in absence or presence of PA28**-**αβ. The values are the average of the SPs measured at different time points. (C) In presence of PA28-αβ, H-carrier 20S immunoproteasomes have a significant lower epitope SP in all three selected ranges of substrate consumption. No significant difference emerged, on the contrary, in absence of PA28-αβ (D). (E) The similar rate constant K_1_ of MBP_102–129_ digestion by 20S immunoproteasomes (in presence of PA28-αβ) carrying the three LMP2 codon 60 variants. All values are given as mean**+**S.E.M. Statistical significance (p<0.05) is marked with *.

We observed that this regulatory complex increased the degradation rate of MBP_102–129_ (Fig. 4A) as well as the MBP_111–119_ specific production (SP) (M-W U = 0; p = 0.002), as also indirectly confirmed by the concomitant higher SP of the flanking peptide 1–9 ( M-W U = 0; p = 0.002) (Fig. 4B).

### LMP2 R60H Polymorphism Influences the Efficiency of MBP_111–119_ Production by 20S Immunoproteasomes in the Presence of PA28-αβ

To investigate the impact of the LMP2 R60H polymorphism on the production of the epitope MBP_111–119_, we performed *in vitro* digestions of the substrate MBP_102–129_ by 20S immunoproteasomes (with or without PA28-αβ) purified from 9 LcLs carrying different LMP2 R60H variants. The epitope SP in the presence of PA28-αβ was associated with the LMP2 R60H polymorphism (F = 15.49; p = 0.004) and was significantly lower (a decrease of 28.8%) in LMP2 H-carriers (t = 8.08; p<0.001) when less than the 25% of the substrate was consumed (Fig. 4C). Such a result was confirmed by a decreased epitope SP of 28.5% and 21.72% when 25–35% (t = 2.97; p = 0.021) or 35–50% (t = 4.75; p = 0.002) of substrate was consumed, respectively. Such an association of the LMP2 R60H polymorphism with the epitope SP was not detected in the absence of PA28-αβ (F = 0.306; p = 0.747, *e.g.*, with 25–35% substrate consumed) and, accordingly, it was similar between LMP2 H-carriers and H-not-carriers when less than 25% (t = −0.47; p = 0.962), 25–35% (t = −0.77; p = 0.462) and 35–50% (t = 0.35; p = 0.744) of the substrate was consumed (Fig. 4D).

Although LMP2 polymorphism influenced the epitope SP, it did not impinge upon the MBP_102–129_ degradation rate either in the absence [22] or in the presence of PA28-αβ as described by the comparison of the degradation rate constants Κ_1_ (Fig. 4E) (F = 0.206; p = 0.820).

### LMP2 60HH Variant Decreases the Risk to Develop MS in HLA-A*02+ Female Population

As *in vitro* experiments showed that LMP2 60H allele decreases the production of the HLA-A*02-restricted MBP_111-119_ epitope we performed a genetic population study to test its possible impact on MS onset. We genotyped two independent samples of 694 and 598 MS patients as well as 845 geographically- and age-matched controls for LMP2 R60H, HLA-A*02 and HLA-DRB1*15 alleles, considering their gender. The impact of an alteration of MBP_111-119_ production on MS pathogenesis should be evident in patients able to present such an epitope on HLA-A*02 MHC-I variant. Accordingly, we compared the LMP2 R60H distribution in HLA-A*02 positive MS patients and control subjects. Both MS population showed the same genetic behavior (Table S1 & S2), hence we grouped them in a MS Italian population of 1262 patients and compared them to the control sample (Table 1). A significant decrease in LMP2 60HH frequency emerged in female MS patients carrying the HLA-A*02 allele (*e.g.* LMP2 60HH vs RR: p  =  0.005; OR  =  0.443; 95% CI  =  0.249 – 0.790) (Table 2) but not in HLA-A*02-positive male MS patients (Table 3) nor in HLA-A*02-negative female and male MS patients (data not shown). In female controls LMP2 60H was not in linkage disequilibrium (LD) neither with HLA-DRB1*15 (D’  =  0.02; r^2^ < 0.001; p > 0.05) nor with HLA-A*02 (D’  =  0.03; r^2^  =  0.001; p > 0.05). Both MS and control populations were in Hardy-Weinberg equilibrium for LMP2 codon 60 polymorphism (data not shown). Furthermore, HLA-A*02 allele was a protective factor against MS (p  =  0.001; OR  =  0.671; 95% CI  =  0.529 - 0.851) while HLA-DRB1*15 allele was a risk factor (p < 0.001; OR  =  2.525; 95% CI  =  1.801 - 3.754) (data not shown), as already reported in the Italian population too [3], [32], [33], [34], [35]. No effects on the MS onset age exerted by LMP2 polymorphism in HLA-A*02-positive female MS population (Table 4) and by HLA-A*02 in the total MS population was observed (Table 5).

**Table 1:** Gender distribution in Italian MS and control populations. Values in brackets in distribution column are percentages. The Italian MS population described in this table is the result of sum of two independent Italian MS samples reciprocally in accordance (see also Tables S1 & S2).

	MS (n = 1262)	control (n = 845)
male	413 (32.7)	437 (51.7)
female	849 (67.3)	408 (48.3)

**Table 2:** LMP2 60HH variant decreases the risk to develop MS in HLA-A*02 carrier female population. Values in brackets in distribution column are percentages. Statistical results are reported for each genetic analysis. The Italian MS female population described in this table is the result of sum of two independent Italian MS samples reciprocally in accordance (see also Tables S1 & S2).

Genotype	MS (n = 337)	control (n = 202)	p	OR (95% CI)
HH	26 (7.7)	32 (15.8)		
RH	146 (43.3)	80 (39.6)	0.013	
RR	165 (49.0)	90 (44.6)		
HH vs RR			0.005	0.443 (0.249–0.790)
HH vs RH			0.006	0.445 (0.248–0.799)
RH vs RR			0.981	0.995 (0.684–1.448)

Allele	MS (n = 674)	control (n = 404)	p	OR (95% CI)
H	198 (29.4)	144 (35.6)	0.032	0.840 (0.713–0.989)
R	476 (70.6)	260 (64.4)		

**Table 3:** LMP2 R60H distribution in HLA-A*02 carrier MS and control male populations. Values in brackets in distribution column are percentages. Statistical results are reported for each genetic analysis. The Italian MS male population described in this table is the result of sum of two independent Italian MS samples reciprocally in accordance (see also Tables S1 & S2).

Genotype	MS (n = 178)	control (n = 194)	p	OR (95% CI)
HH	18 (10.1)	21 (10.8)		
RH	85 (47.8)	88 (45.4)	0.281	
RR	75 (42.1)	85 (43.8)		
HH vs RR			0.728	0.868 (0.389–1.933)
HH vs RH			0.948	1.026 (0.468–2.248)
RH vs RR			0.501	0.846 (0.518–1.379)

Allele	MS (n = 356)	control (n = 388)	p	OR (95% CI)
H	121 (34.0)	130 (33.5)	0.579	0.908 (0.646–1.276)
R	235 (66.0)	258 (66.5)		

**Table 4:** LMP2 R60H polymorphism and MS onset age in HLA-A*02 carrier female MS population. Means and standard deviations (SD) are reported. The Italian HLA-A*02 carrier female MS population described in this table is the result of sum of two independent Italian MS samples reciprocally in accordance (see also Tables S1 & S2).

Genotype	mean (years)	SD	p
HH	32.61	7.65	0.329
RH	29.48	9.65	
RR	30.65	10.57	

**Table 5:** MS onset age in HLA-A*02 carrier and not-carrier MS populations. Means and standard deviations (SD) are reported. The Italian MS population described in this table is the result of sum of two independent Italian MS samples reciprocally in accordance (see also Tables S1 & S2).

HLA-A*	mean (years)	SD	p
02 not-carriers	31.11	10.79	0.206
02 carriers	30.27	10.21	

## Discussion

The observed gender-dependency of the genetic association is consistent with other studies describing a gender dimorphism in cytokine response towards myelin antigens and a hormonal regulation of the immune system in MS [36], [37], [38]. LMP2 R60H polymorphism association is gender- (male-) dependent in another autoimmune disease, *i.e.* dermal psoriasis [9]. The bulk of these indirect observations suggest a connection between LMP2 codon 60 polymorphism, immunoproteasome activity and gender in immunological dysfunctions. Despite the scarcity of experimental evidences we might speculate that the cytokines responses towards MHC-I myelin epitopes (as demonstrated for the MHC-II [38]) is altered by gender, generating a scenario in which the influence of the LMP2 R60H polymorphism on specific myelin epitopes might impinges upon the MS onset only in females. Indeed, the decreased risk of female subjects carrying the HLA-A*02 allele and the LMP2 60HH variant to develop MS might be explained by the impact of the LMP2 polymorphism on the generation of the HLA-A*02-restricted epitope (MBP_111–119_) as well of other epitopes not investigated in this study. Indeed, the decreased presentation of this epitope on the MHC-I (HLA-A*02) complex of antigen presenting cells or other cells involved in MS pathways could reduce the probability to disrupt the physiological tolerance (central or peripheral) of MBP-reactive CD8+ T cells and/or their cytotoxicity towards oligodendrocytes thereby restraining the MS onset. We showed that the main producers of myelin sheets, *i.e.* the oligodendrocytes, as well as the macrophages/microglial cells, which express PA28-αβ [39] and golli MBP [40] during EAE, are positive to LMP2 and PA28-αβ staining in MS CNS (Fig. 3). In oligodendrocytes, a decreased presentation mediated by the LMP2 60HH variant of MBP epitopes (*e.g.* MBP_111–119_) on HLA-A*02 MHC could attenuate the cytotoxic activity of MBP-reactive CD8+ T cells against their main cell targets. In macrophages/microglial cells (Fig. 3), the lower presentation of these epitopes could restrain a further clonal expansion of self-reactive CD8+ T cells, which has been assumed as one of the key steps of MS development [1]. Taking into account that CNS endothelial cells express immunoproteasomes and PA28-αβ (Fig. 1) as well as they can present MBP in animal models [41], [42], [43], the LMP2 60HH-dependent altered expression of the MBP epitopes (*e.g.* MBP_111–119_) might also affect the blood-brain barrier (BBB) crossing. Indeed, it has been recently proposed that, after priming, activated CD8+ T cells cross the BBB by an antigen-specific interaction with cerebral endothelium [44]. Thus, endothelial cells carrying HLA-A*02 and LMP2 60HH might lead to a decreased BBB crossing by MBP-reactive CD8+ T cells during the early phases of neuroinflammation, affecting MS development. Furthermore, LMP2 R60H polymorphism might influence not only the mechanisms leading to MS that occur in CNS but also the complex balance between central and peripheral tolerance of MBP-restricted CD8+ T cells and thereby their pathological activation in peripheral lymph nodes (*e.g.* cervical and lumbal) by dendritic cells [1]. Indeed, these cells, which express immunoproteasomes and PA28-αβ at their mature stage [45], play a key role in the thymic selection and in peripheral activation of MBP-restricted CD8+ T cells by the presentation of endogenous MBP on MHC-I [28], [29]. As consequence, an alteration of the amount of MBP epitopes (*e.g.* MBP_111–119_) presented on dendritic cells due to the LMP2 polymorphism might affect the repertoire of MBP-reactive CD8+ T cells and, thereby their eventual activation during the early phases of MS onset.

Our investigation also addresses the complex role that HLA-A*02 appears to have in MS. We observed a decreased risk to develop MS in Italian female population carrying the HLA-A*02 allele, in agreement with other studies [32], [33], [46]. Although the mechanisms are still unknown, HLA-A*02 variant in a specific EAE model appeared to increase the negative selection of MBP-reactive CD8+ thymocytes and to decrease the responsiveness of MBP-reactive CD8+ T cells in periphery [4], thereby protecting from EAE. Our results suggest that LMP2 60HH variant impinges upon the likelihood to develop MS but only in HLA-A*02 carriers. Likewise, LMP2 60H allele decreases the *in vitro* generation of the MBP_111–119_ epitope by immunoproteasomes thereby suggesting that this specific myelin epitope could play a pathogenetic role in MS when presented on HLA-A*02 MHC. Accordingly, several CD8+ T cells reactive to HLA-A*02 myelin epitopes have been identified in peripheral blood of MS subjects [14]. In particular, MBP_111–119_–reactive CD8+ T cells, which are increased three fold in MS patients peripheral blood, are CD45RO+ memory T cells secreting TNF-α and IFN-γ after epitope challenging and are cytotoxic towards antigen presenting cells and oligodendrocytes, thus supporting the postulate that these CD8+ T cells could contribute to the tissue injury in MS [13], [15].

In conclusion, our data suggests that the presence of immunoproteasomes in the CNS could be a marker of a pathological scenario involving neuroinflammation (autoimmunity, neurodegeneration but also ageing) [6], [7], [47], therefore putting immunoproteasome forward as potential candidate for future therapeutic approaches [48], [49], [50], [51], [52].

## Supporting Information

Figure S1 MBP_111–119_ binds the HLA-A*0201 complex with relative good affinity. A) Comparison of the amount of HLA-A*0201 complexes (marked with a FITC-Ab) presented on the outer surface of T2 cells treated with 100 µg/ml MBP_111–119_ or (ELA)-MART1_26–35_ epitopes. In particular, signals of T2 cells are reported: i. without FITC-Ab and epitope; ii. without epitopes and with FITC-Ab; iii. with FITC-Ab and MBP_111–119_ epitope; iv. with FITC-Ab and (ELA)-MART1_26–35_ epitope. B) The chart shows the binding affinity of the peptides for the MHC class I complexes presented to the outer surface of T2 cells. The net fluorescence is the fluorescence's difference between the cells incubated with and without the epitopes.
(2.00 MB TIF)

Table S1 LMP2 60HH, HLA-A*02 and gender frequency in the first investigated Italian MS population (n = 694) compared to age-matched Italian control population (n = 845). (A) Gender distribution in MS patients and control populations. (B) LMP2 R60H polymorphism and allele distribution in MS and control HLA-A*02 carrier populations, taking into account the gender. (C) LMP2 R60H polymorphism and MS onset age in HLA-A*02-positive female MS patients. Values in brackets in distribution column are percentages. Statistical results are reported for each genetic analysis.
(0.14 MB DOC)

Table S2 LMP2 60HH, HLA-A*02 and gender frequency in the second investigated Italian MS population (n = 598) compared to age-matched Italian control population (n = 845). (A) Gender distribution in MS patients and control populations. (B) LMP2 R60H polymorphism and allele distribution in MS and control HLA-A*02 carrier populations, taking into account the gender. (C) LMP2 R60H polymorphism and MS onset age in HLA-A*02-positive female MS patients. Values in brackets in distribution column are percentages. Statistical results are reported for each genetic analysis.
(0.14 MB DOC)
