# Supplementary figures and images for: Immunoproteasome LMP2 60HH Variant Alters MBP Epitope Generation and Reduces the Risk to Develop Multiple Sclerosis in Italian Female Population
Source: PLoS One. 2010 Feb 18;5(2):e9287. doi: 10.1371/journal.pone.0009287 (PMC2823778; doi:10.1371/journal.pone.0009287)

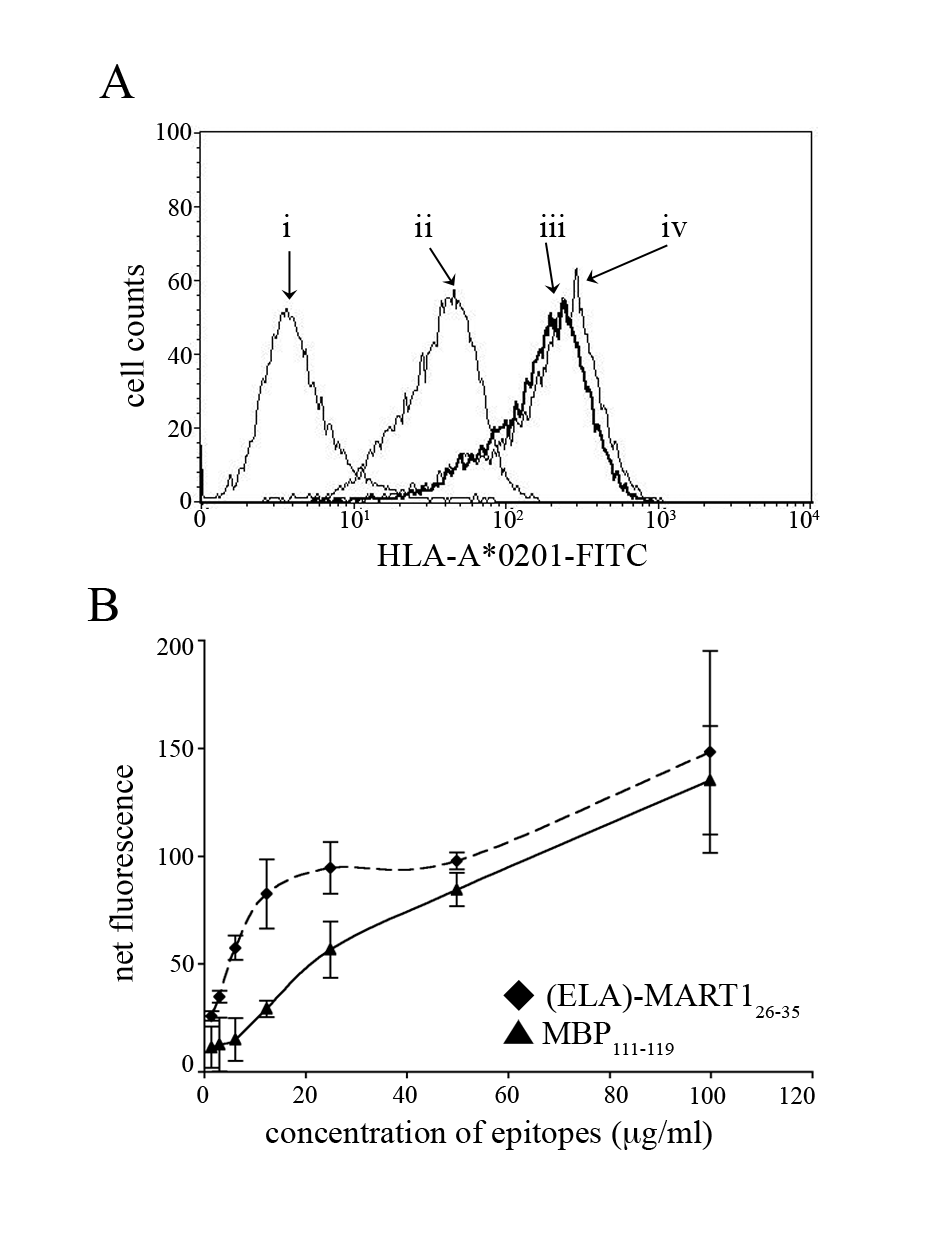

Supplement: Figure S1 — MBP111–119 binds the HLA-A*0201 complex with relative good affinity. A) Comparison of the amount of HLA-A*0201 complexes (marked with a FITC-Ab) presented on the outer surface of T2 cells treated with 100 µg/ml MBP111–119 or (ELA)-MART126–35 epitopes. In particular, signals of T2 cells are reported: i. without FITC-Ab and epitope; ii. without epitopes and with FITC-Ab; iii. with FITC-Ab and MBP111–119 epitope; iv. with FITC-Ab and (ELA)-MART126–35 epitope. B) The chart shows the binding affinity of the peptides for the MHC class I complexes presented to the outer surface of T2 cells. The net fluorescence is the fluorescence's difference between the cells incubated with and without the epitopes. (2.00 MB TIF) [file pone.0009287.s001.tif]
