# Supplementary material for: Immunoproteasome LMP2 60HH Variant Alters MBP Epitope Generation and Reduces the Risk to Develop Multiple Sclerosis in Italian Female Population
Source: PLoS One. 2010 Feb 18;5(2):e9287. doi: 10.1371/journal.pone.0009287 (PMC2823778; doi:10.1371/journal.pone.0009287)
Supplement: Table S1 — LMP2 60HH, HLA-A*02 and gender frequency in the first investigated Italian MS population (n = 694) compared to age-matched Italian control population (n = 845). (A) Gender distribution in MS patients and control populations. (B) LMP2 R60H polymorphism and allele distribution in MS and control HLA-A*02 carrier populations, taking into account the gender. (C) LMP2 R60H polymorphism and MS onset age in HLA-A*02-positive female MS patients. Values in brackets in distribution column are percentages. Statistical results are reported for each genetic analysis. (0.14 MB DOC) [file pone.0009287.s002.doc]

|  |  |  |
| --- | --- | --- |
|  |  |  |
|  |  |  |

|  |  |  |  |  |
| --- | --- | --- | --- | --- |
|  |  |  |  |  |
|  |  |  |  |  |
|  |  |  |  |  |
|  |  |  |  |  |
|  |  |  |  |  |
|  |  |  |  |  |
|  |  |  |  |  |
|  |  |  |  |  |
|  |  |  |  |  |
|  |  |  |  |  |
|  |  |  |  |  |
|  |  |  |  |  |
|  |  |  |  |  |
|  |  |  |  |  |
|  |  |  |  |  |
|  |  |  |  |  |
|  |  |  |  |  |
|  |  |  |  |  |
|  |  |  |  |  |
|  |  |  |  |  |
|  |  |  |  |  |
|  |  |  |  |  |

Gender distribution in MS and control populations.

|  |  |  |
| --- | --- | --- |
|  |  |  |
|  |  |  |

| A) Gender distribution in MS and control populations | | | | | |
| --- | --- | --- | --- | --- | --- |
|  | | | | | |
|  | **MS (n=694)** | **control (n=845)** |  |  | |
| male | 230 (33.1) | 437 (51.7) |  |  | |
| female | 464 (66.9) | 408 (48.3) |  |  | |
|  |  |  |  |  | |
| B) LMP2 R60H polymorphism distribution in MS and control HLA-A*02 carrier populations | | | | | |
|  | | | | | |
|  | **Female** |  |  |  |  |
| **Genotype** | **MS (n=188)** | **control (n=202)** | **p** | **OR (95% CI)** |  |
| HH | 15 (8.0) | 32 (15.8) | 0.039 |  |  |
| RH | 73 (38.8) | 80 (39.6) |  |  |  |
| RR | 100 (53.2) | 90 (44.6) |  |  |  |
| HH vs RR |  |  | 0.010 | 0.42 (0.20-0.87) |  |
| HH vs RH |  |  | 0.056 | 0.51 (0.24-1.07) |  |
| RH vs RR |  |  | 0.370 | 0.82 (0.52-1.29) |  |
| **Allele** | **MS (n=376)** | **control (n=404)** | **p** | **OR (95% CI)** |  |
| H | 103 (27.4) | 144 (35.6) | 0.013 | 0.68 (0.50-0.93) |  |
| R | 273 (72.6) | 260 (64.4) |  |  |  |
|  |  |  |  |  |  |
|  | **Male** |  |  |  |  |
| **Genotype** | **MS (n=96)** | **control (n=194)** | **p** | **OR (95% CI)** |  |
| HH | 11 (11.5) | 21 (10.8) | 0.880 |  |  |
| RH | 46 (47.9) | 88 (45.4) |  |  |  |
| RR | 39 (40.6) | 85 (43.8) |  |  |  |
| HH vs RR |  |  | 0.750 | 1.14 (0.45-2.77) |  |
| HH vs RH |  |  | 1.000 | 1.00 (0.40-2.40) |  |
| RH vs RR |  |  | 0.620 | 1.14 (0.66-2.00) |  |
| **Allele** | **MS (n=192)** | **control (n=388)** | **p** | **OR (95% CI)** |  |
| H | 68 (35.4) | 130 (33.5) | 0.650 | 1.09 (0.74-1.59) |  |
| R | 124 (64.6) | 258 (66.5) |  |  |  |
|  |  |  |  |  | |
| C) LMP2 R60H polymorphism and MS onset age in HLA-A*02+ female MS population | | | | | |
|  | | | | | |
| **Genotype** | **mean (years)** | **SD** | **p** |  | |
| HH | 32.25 | 8.74 | 0.690 |  | |
| RH | 30.10 | 9.41 |  |  | |
| RR | 29.61 | 10.37 |  |  | |

C) LMP2 R60H polymorphism and MS onset age in HLA-A*02+ female MS population.

|  |  |  |  |
| --- | --- | --- | --- |
|  |  |  |  |
|  |  |  |  |
|  |  |  |  |
