# Supplementary material for: Immunoproteasome LMP2 60HH Variant Alters MBP Epitope Generation and Reduces the Risk to Develop Multiple Sclerosis in Italian Female Population
Source: PLoS One. 2010 Feb 18;5(2):e9287. doi: 10.1371/journal.pone.0009287 (PMC2823778; doi:10.1371/journal.pone.0009287)
Supplement: Table S2 — LMP2 60HH, HLA-A*02 and gender frequency in the second investigated Italian MS population (n = 598) compared to age-matched Italian control population (n = 845). (A) Gender distribution in MS patients and control populations. (B) LMP2 R60H polymorphism and allele distribution in MS and control HLA-A*02 carrier populations, taking into account the gender. (C) LMP2 R60H polymorphism and MS onset age in HLA-A*02-positive female MS patients. Values in brackets in distribution column are percentages. Statistical results are reported for each genetic analysis. (0.14 MB DOC) [file pone.0009287.s003.doc]

|  |  |  |
| --- | --- | --- |
|  |  |  |
|  |  |  |

|  |  |  |  |  |
| --- | --- | --- | --- | --- |
|  |  |  |  |  |
|  |  |  |  |  |
|  |  |  |  |  |
|  |  |  |  |  |
|  |  |  |  |  |
|  |  |  |  |  |
|  |  |  |  |  |
|  |  |  |  |  |
|  |  |  |  |  |
|  |  |  |  |  |
|  |  |  |  |  |
|  |  |  |  |  |
|  |  |  |  |  |
|  |  |  |  |  |
|  |  |  |  |  |
|  |  |  |  |  |
|  |  |  |  |  |
|  |  |  |  |  |
|  |  |  |  |  |
|  |  |  |  |  |
|  |  |  |  |  |
|  |  |  |  |  |

Gender distribution in MS and control populations.

|  |  |  |
| --- | --- | --- |
|  |  |  |
|  |  |  |

| A) Gender distribution in MS and control populations | | | | | |
| --- | --- | --- | --- | --- | --- |
|  | | | | | |
|  | **MS (n=568)** |  |  |  | |
| male |  |  |  |  | |
| female |  |  |  |  | |
|  |  |  |  |  | |
| B) LMP2 R60H polymorphism distribution in MS and control HLA-A*02 carrier populations | | | | | |
|  | | | | | |
|  | **Female** |  |  |  |  |
| **Genotype** | **MS (n=149)** | **control (n=202)** | **p** | **OR (95% CI)** |  |
| HH | 11 (7.4) | 32 (15.8) | 0.034 |  |  |
| RH | 73 (49.0) | 80 (39.6) |  |  |  |
| RR | 65 (43.6) | 90 (44.6) |  |  |  |
| HH vs RR |  |  | 0.051 | 0.48 (0.20-1.06) |  |
| HH vs RH |  |  | 0.009 | 0.38 (0.16-0.84) |  |
| RH vs RR |  |  | 0.308 | 1.26 (0.80-2.03) |  |
| **Allele** | **MS (n=298)** | **control (n=404)** | **p** | **OR (95% CI)** |  |
| H | 95 (31.9) | 144 (35.6) | 0.300 | 0.84 (0.61-1.17) |  |
| R | 203 (68.1) | 260 (64.4) |  |  |  |
|  |  |  |  |  |  |
|  | **Male** |  |  |  |  |
| **Genotype** | **MS (n=82)** | **control (n=194)** | **p** | **OR (95% CI)** |  |
| HH | 7 (8.5) | 21 (10.8) | 0.836 |  |  |
| RH | 39 (47.6) | 88 (45.4) |  |  |  |
| RR | 36 (43.9) | 85 (43.8) |  |  |  |
| HH vs RR |  |  | 0.620 | 0.80 (0.26-2.14) |  |
| HH vs RH |  |  | 0.550 | 0.75 (0.25-2.04) |  |
| RH vs RR |  |  | 0.870 | 1.05 (0.59-1.88) |  |
| **Allele** | **MS (n=164)** | **control (n=388)** | **p** | **OR (95% CI)** |  |
| H | 53 (32.3) | 130 (33.5) | 0.800 | 0.95 (0.63-1.42) |  |
| R | 111 (67.7) | 258 (66.5) |  |  |  |
|  |  |  |  |  | |
| C) LMP2 R60H polymorphism and MS onset age in HLA-A*02+ female MS population | | | | | |
|  | | | | | |
| **Genotype** | **mean (years)** | **SD** | **p** |  | |
| HH | 33.00 | 6.66 | 0.179 |  | |
| RH | 28.91 | 9.91 |  |  | |
| RR | 31.95 | 10.77 |  |  | |

C) LMP2 R60H polymorphism and MS onset age in HLA-A*02+ female MS population.

|  |  |  |  |
| --- | --- | --- | --- |
|  |  |  |  |
|  |  |  |  |
|  |  |  |  |
